# Pharmacogenetic evaluation to assess breakthrough psychosis with aripiprazole long-acting injection: a case report

**DOI:** 10.1186/s12888-017-1396-x

**Published:** 2017-07-03

**Authors:** Seenae Eum, Mark E. Schneiderhan, Jacob T. Brown, Adam M. Lee, Jeffrey R. Bishop

**Affiliations:** 1 College of Pharmacy, Department of Experimental and Clinical Pharmacology, University of Minnesota, 308 Harvard St. S.E., Minneapolis, MN 55455 USA; 2 College of Pharmacy, Department of Pharmacy Practice and Pharmaceutical Sciences, University of Minnesota, 1110 Kirby Dr., Duluth, MN 55812 USA; 3 College of Pharmacy, Department of Experimental and Clinical Pharmacology and College of Medicine, Department of Psychiatry, University of Minnesota, 308 Harvard St. S.E., Minneapolis, MN 55455 USA

**Keywords:** Aripiprazole long-acting injectable, Wearing-off symptoms, *DRD2*, Clinical pharmacogenetic testing

## Abstract

**Background:**

Given the complex nature of symptom presentation and medication regimens, psychiatric clinics may benefit from additional tools to personalize treatments. Utilizing pharmacogenetic information may be helpful in assessing unique responses to therapy. We report herein a case of wearing-off phenomena during treatment with aripiprazole long-acting injectable (LAI) and a proof of concept strategy of how pharmacogenetic information may be used to assess possible genetic factors and also hypothesize potential mechanisms for further study.

**Case presentation:**

A 51-year-old African American male with schizoaffective disorder was referred to a psychiatric clinic for medication management. After unsuccessful trials of multiple antipsychotics, oral aripiprazole was initiated (up to 30 mg/day) and transitioned to aripiprazole LAI with symptom improvement. At a high dose of aripiprazole LAI (400 mg Q3wks), the patient experienced breakthrough symptoms approximately 3 days prior to his next injection. Various considerations were examined to explain his atypical dose requirements, including but not limited to pharmacogenetic influences. Pharmacogenetic testing ruled out genetic influences on drug metabolism but noted a -141C Del variant in the dopamine-D2 receptor (*DRD2*) gene associated in prior studies of poor-response to antipsychotics. At this time, a new formulation, aripiprazole lauroxil, was explored due to its availability in higher dose options. Transition to the new formulation (882 mg Q4wks) greatly improved and stabilized the patient’s symptoms with no breakthrough psychosis. Comparable daily dose equivalents were achieved with two different formulations due to the Q3wks vs Q4wks dosing strategies, although the two agents have some differences in pharmacokinetic profiles.

**Conclusions:**

We report a case of a patient experiencing wearing-off symptoms with aripiprazole LAI who benefited from switching to aripiprazole lauroxil. Pharmacogenetic testing revealed normal activity for relevant metabolism pathways but a *DRD2* -141C variant that may influence brain D2 expression and antipsychotic responsiveness. The clinical utility of *DRD2* information and what to do with genotyping results has not been previously addressed, despite availability on clinical test panels. Our case report suggests further investigations of altered dosing strategies and receptor genotype sensitivities to pharmacokinetic factors may be helpful in understanding symptom re-emergence observed in some patients taking LAI antipsychotics.

## Background

Approximately one-third of patients with schizophrenia treated with antipsychotics continue to experience persistent psychotic symptoms [1]. Moreover, given the complex nature of symptom presentation and medication regimens in patients with psychotic disorders, outpatient psychiatric clinics are in need of ways to improve patient care. Although there are no simple tests to determine if a patient will have a response or remission of schizophrenia symptoms with a selected antipsychotic agent and dose, pharmacogenetic information along with other clinical information may offer great potential to optimize pharmacotherapy and improve the overall quality of life in psychiatric patients.

Pharmacogenetics is the study of how genetic variations influence drug therapy and focuses on the variants associated with pharmacokinetics and pharmacodynamics of medications. Over 160 medications, including antipsychotics such as aripiprazole, list pharmacogenetic information in the product labeling to date [2]. Neuropsychiatric medications account for about one-third of those, including many commonly used antidepressant, antipsychotic, and antiepileptic medications [3]. While research in psychiatric pharmacogenetics has significantly increased over the past 20 years, clinical application is just beginning with many emerging questions of how, when, and if to use this information.

The pharmacogenetic biomarker most commonly included in the product labeling for many antipsychotic medications is the cytochrome P450 2D6 (*CYP2D6*) gene related to drug metabolism and pharmacokinetic properties of drugs such as aripiprazole, risperidone, perphenazine, iloperidone, and others [2, 4, 5]. Genes thought to impact pharmacodynamics of antipsychotics (e.g. dopamine-D2, serotonin-5HT2A receptors, etc.) are not included in product labeling, but are often included in many available genetic testing panels for psychotropic medications. While there are studies published highlighting the mechanistic consequences of some polymorphisms in these genes as well as associations with some clinical outcomes, the clinical utility of pharmacodynamic gene variants in guiding antipsychotic pharmacotherapy is not as clear as variants which influence pharmacokinetics. Variation in the dopamine-D2 receptor gene (*DRD2*) is one example of a pharmacodynamic gene that has been extensively investigated in relation to treatment outcomes, as it is a primary target for all currently available antipsychotic agents. Although there are conflicting results, meta-analyses highlight that the rs1799732 -141C Ins/Del ‘deletion’ polymorphism may be associated with decreased responsiveness to antipsychotic medications [6, 7]. How to consider this information in the context of clinical care is unclear.

Aripiprazole (oral), one of the antipsychotic agents examined in the meta-analysis [6], was approved by the U.S. Food and Drug Administration (FDA) in late 2002 for the treatment of schizophrenia, and is a unique second generation antipsychotic with partial agonist activity at D2 and serotonin (5-hydroxytryptamine, 5-HT) 1A receptors, and antagonist activity at 5-HT2A receptors [8]. The first aripiprazole long-acting injectable (LAI) formulation, Abilify Maintena®, was approved in early 2013 at the recommended dose of 400 mg IM injection every 4 weeks [8]. Aripiprazole lauroxil (Aristada®) is a newer LAI aripiprazole formulation, which was FDA approved in October 2015 [8]. Aripiprazole lauroxil is an N-acyloxymethyl prodrug that undergoes a 2-step bioconversion in the plasma from the lauroxil to an intermediate, N-hydroxymethyl-aripiprazole via enzyme-mediated hydrolysis. The N-hydroxymethyl-aripiprazole then undergoes a hydrolysis reaction to aripiprazole [9]. Aripiprazole lauroxil is available at a dose of 441 mg (deltoid or gluteal), 662 mg and 882 mg (only gluteal) corresponding to aripiprazole LAI 300 mg, 450 mg and 600 mg (corresponding to oral aripiprazole 10 mg/day, 15 mg/day and 20 mg/day), respectively [8]. While these two formulations are similar with respect to dosing/administration intervals, they are different with regards to their formulations which influence some of their pharmacokinetic parameters including T_1/2_ and T_max_ [8]. The clinical implications of these differences in the context of altered drug metabolism or target receptor sensitivities are unclear.

The dosing strategies for LAI antipsychotics include relatively standardized conversions from oral formulations to recommended administration intervals. The injection intervals for LAIs depend on the specific agent (e.g. every 4 weeks for aripiprazole, haloperidol, and paliperidone; every 2 weeks for risperidone, etc.) and are based on extensive pharmacokinetic studies of patients as well as computer simulations [10]. Although not extensively reported in the literature, there are clinical reports of some patients requiring shorter dosing intervals or higher than approved doses of LAI antipsychotics with unclear reasons.

Here, we present a case of a patient with treatment-refractory schizoaffective disorder for whom pharmacogenetic testing was obtained in an attempt to explain his wearing-off symptoms with aripiprazole LAI 400 mg every 3 weeks combined with daily oral aripiprazole. Although there have been anecdotal clinical reports of symptom breakthrough at the end of a LAI antipsychotic dosing cycle, no published studies or cases have previously reported this phenomena with aripiprazole LAI. This case discussion focuses on pharmacogenetic testing as a proof of concept strategy to hypothesize a mechanism and management strategy for these breakthrough phenomena with aripiprazole LAI.

## Case presentation

AB, a 51-year-old African American male [height 195.6 cm, weight 141 kg, body mass index 36.9 kg/m^2^] with chart diagnoses of schizoaffective disorder, depressive type and post-traumatic stress disorder, was referred to a community mental health center in March of 2014 to address medication non-adherence and lack of medication effectiveness. AB’s symptoms were first reported in 2006 and included depressed mood and persecutory auditory hallucinations with command hallucinations to either self-harm (e.g. insulin overdose) or stop his medications. His referral in 2014 included details of treatment and symptoms since 2008. The referral notes highlighted a complicated psychiatric history with approximately eight psychiatric hospitalizations over the previous five years, predominantly attributed to depressive episodes and suicide attempts. His other medical history includes uncontrolled type 2 diabetes with diabetic neuropathy, hypertension, asthma, obstructive sleep apnea, diverticulitis, chronic musculoskeletal pain, and chronic headaches. His medication list at the time of this report (February, 2017) is provided in Table 1.

**Table 1:** Patient’s current medication list

Indication	Medication	Dose	Route of Administration
Mental Health			
Schizoaffective disorder	Aripiprazole lauroxil	882 mg once a month	IM
Depression	Sertraline	150 mg every morning	PO
Insomnia	Zolpidem CR	12.5 mg at bedtime	PO
Smoking cessation	Varenicline	1 mg BID	PO
Cardiovascular			
Hypertension	Amlodipine	10 mg once a day	PO
	Atenolol	50 mg once a day	PO
	Losartan	100 mg once a day	PO
	Spironolactone	100 mg once a day	PO
Prevention of CV disease	Aspirin	81 mg once a day	PO
Type 2 DM			
DM	Insulin glargine	120 units BID	SC
	Insulin aspart	As directed	SC
	Metformin	1000 mg BID	PO
Diabetic neuropathy	Gabapentin	1200 mg TID	PO
Respiratory			
Asthma	Ipratropium-albuterol	18–103 mcg 2 puffs BID	Nasal
	Albuterol nebulizer	As directed	Nasal
	Budesonide-formoterol	160–4.5 mcg 2 puffs BID	Nasal
Congestion	Fluticasone	50 mcg 1 spray once a day	Nasal
	Oxymetazoline	0.05% 1 spray every night	Nasal
Pain			
Pain	Tramadol	100 mg TID PRN	PO
Gastrointestinal			
GERD	Omeprazole	20 mg BID	PO

*IM* intramuscular, *PO* by mouth, *CR* controlled release, *BID* twice a day, *CV* cardiovascular, *DM* diabetes mellitus, *SC* subcutaneous, *TID* three times a day, *PRN* as needed, *GERD* gastroesophageal reflux disease

Between 2008 and 2012, he was trialed on and off multiple antipsychotic agents including risperidone (titrated up to 6 mg/day), haloperidol (titrated up to 15 mg/day), quetiapine (titrated up to 800 mg/day), and ziprasidone (titrated up to 200 mg/day); all for a duration of at least 12 weeks without significant improvement. A trial of olanzapine (titrated up to 15 mg/day) resulted in slightly improved psychotic symptoms; however, was ultimately discontinued due to worsening diabetes control. At this time, oral aripiprazole 5 mg/day was started (April 2012).

At the time of his referral (March, 2014), the patient was on oral aripiprazole 15 mg/day, which was titrated up to 30 mg/day through March 2015, and the patient reported improvement in auditory hallucinations, mood, and suicidal ideations. In March 2015, AB agreed to start long-acting injectable (LAI) aripiprazole with the goal to eventually discontinue the oral aripiprazole to provide a safeguard for non-adherence if the command hallucinations returned. Subsequently, aripiprazole LAI, 400 mg intramuscularly (IM) was administered into the gluteal muscle every 4-weeks combined with oral aripiprazole 30 mg/day. The plan was to reduce the aripiprazole oral dose to 15 mg/day at 2-weeks post-injection. Eight weeks after initiating aripiprazole LAI, a marked improvement in psychiatric symptoms was noted. Over approximately 12-weeks, the oral aripiprazole supplementation was reduced to 5 mg/day (June 2015); however, within 4-weeks of oral aripiprazole dose reduction to 5 mg/day, his auditory hallucinations returned and worsened. The oral aripiprazole dose was then increased back to 7.5 mg/day with a noticeable positive response. At a 2-week follow-up appointment after this dose increase (July 2015), auditory hallucinations were reported to be “not as bad” but worse 1-week prior to his aripiprazole injection, resulting in a modification of aripiprazole LAI frequency from 400 mg IM every 4-weeks to every 3-weeks along with the 7.5 mg/day oral aripiprazole. In October 2015, since he complained of worsening auditory hallucinations approximately 3 days prior to his next injection, oral aripiprazole 2 mg/day as needed (up to 2 times/week) was added to his current regimen (aripiprazole LAI 400 mg every 3-week and oral aripiprazole 7.5 mg/day) for this wearing-off symptoms.

The continuing need for oral aripiprazole and symptom breakthrough prior to his every 3-week aripiprazole injection prompted further inquiry as to which variables might be contributing to this pattern of response/non-response. Considerations included medications/substances interactions, pharmacokinetic characteristics, and administration techniques (i.e. site of administration, syringe length and gauge, etc.), all of which were ruled out as contributing factors. No significant drug/substance interactions were noted (Table 1). Since adherence was not in question with an LAI medication, serum drug concentrations were not ordered. Pharmacogenetic variables influencing drug metabolism were then considered, and a commercially available psychiatry-focused pharmacogenetic testing panel was ordered in October 2015 (Table 2).

**Table 2:** Patient’s pharmacogenetic test results

Gene	Genotype	Predicted Phenotype
*CYP2C19*	*1/*2	Intermediate Metabolizer
*CYP2D6*	*1/*2	Extensive (Normal) Metabolizer
*CYP3A4*/*CYP3A5*	*1/*1; *1/*1	Extensive (Normal) Metabolizer
*UGT2B15*	*1/*1	Extensive (Normal) Metabolizer
*DRD2*	INS/DEL	Reduced Responder
*HTR2C*	C/C	Normal Expressor
*MTHFR*	C/C (C677T); A/A (A1298C)	Normal Activity

*CYP* cytochromes P450, *UGT* uridine 5′-diphospho-glucuronosyltransferase, *DRD2* dopamine receptor D2 gene, *HTR2C* 5-hydroxytryptamine receptor 2C gene, *MTHFR* methylene tetrahydrofolate reductase gene

Pharmacogenetic test results for drug-metabolizing enzymes cytochrome P450 2D6 (*CYP2D6*) and *CYP3A4* did not explain the patient’s need for high aripiprazole dose nor his breakthrough symptoms. However, the patient was found to carry a genetic variation in the dopamine receptor D2 gene (*DRD2*), −141C Ins/Del polymorphism (rs1799732). This polymorphism has been linked to variation in striatal dopamine receptor density [11, 12] and reduced response to some antipsychotics [6], which is consistent with AB’s reduced response to many antipsychotic agents.

With no clear pharmacogenetic influence on drug metabolism, yet a documented need for higher doses of aripiprazole, additional dosing strategies were explored. At this time, a new long-acting injectable formulation of aripiprazole, aripiprazole lauroxil, was considered due higher dosing options (aripiprazole lauroxil 882 mg every 4-weeks, equivalent to aripiprazole LAI 600 mg) [8, 13]. Therefore, in December 2015, the decision was made to switch to aripiprazole lauroxil 882 mg IM (gluteal) every 4-weeks. Although the plan was to continue the oral aripiprazole for 3 weeks, the patient discontinued the oral aripiprazole on his own after the first injection. The patient initially noted injection site pain with the lauroxil formulation after the first 2 administrations, but no other adverse reactions were reported. Due to the every 3-weeks (aripiprazole LAI) vs. every 4-weeks (aripiprazole lauroxil) dosing strategies, the approximate daily doses from two long acting formulations were similar (19 mg/day with aripiprazole LAI vs. 21 mg/day with aripiprazole lauroxil, respectively). As of February 2017, he continues to tolerate the aripiprazole lauroxil and reports not needing to use oral aripiprazole.

## Discussion

Here, we report a case of an African American male experiencing breakthrough symptoms towards the end of the LAI cycle at a high dose of aripiprazole LAI, even in combination with additional oral supplementation. This patient benefited from switching to the lauroxil formulation. Pharmacogenetic testing revealed that the patient is a CYP2D6 normal metabolizer but carries a -141C Ins/Del polymorphism in *DRD2* gene.

In the present case, after multiple antipsychotic failures, aripiprazole therapy has been beneficial in reducing depression, psychotic symptoms, and self-injurious behaviors, but patterns of symptom re-emergence resulted in efforts to determine how best to optimize therapy. Several factors related to AB’s increased aripiprazole dose requirements and breakthrough symptoms were assessed, including medication/substance interactions, injection technique (i.e. length and gauge of the needle and site of administration), and pharmacokinetic characteristics (i.e. large volume of distribution and BMI). Although previous studies suggest that sites of injection as well as injection techniques may impact the pharmacokinetics of other LAI antipsychotics [14, 15], we confirmed that aripiprazole LAI was administered according to the FDA labeling recommendations [8] to rule out improper injection technique or location as a source of dosing/administration variability. In addition, no other factors were found to possibly contribute to his patterns of response.

Aripiprazole is primarily metabolized by CYP3A4 and CYP2D6. Diminished CYP2D6 activity due to reduced or non-functional alleles results in an 80% increase in aripiprazole AUC and 2-fold increase in the elimination half-life in CYP2D6 poor metabolizers (PMs) as compared to extensive metabolizers (EMs), leading to a recommendation to start at a lower dose in CYP2D6 PMs [8, 16]. CYP2D6 ultra-rapid metabolizers (UMs) have increased activity due to functional allele duplications as compared to those who are EMs, and although it is presumed these patients may experience lower levels of aripiprazole and increased concentrations of the CYP2D6 metabolite dehydroaripiprazole, there is a dearth of literature related to this topic. Initially, we hypothesized that this patient may be an UM for CYP2D6 due to his atypical dose requirements; however, this was ruled out with pharmacogenetic testing.

Interestingly the pharmacogenetic test report noted that the patient carries a polymorphism in the *DRD2* gene (−141C Ins/Del, rs1799732). This polymorphism is located in the promoter region of *DRD2* and shown to influence D2 expression [11, 12]. A number of candidate gene studies have investigated the relationship between the -141C Ins/Del variant and susceptibility to schizophrenia, producing conflicting results [17, 18]. Other genetic studies have identified over 4000 variants in *DRD2*, some of which were shown to be related to risk for psychiatric disorders in genome-wide association studies (GWAS) [19]. A meta-analyses of 6 studies identified an association of the -141C Del polymorphism with poorer clinical response to antipsychotic agents (risperidone, olanzapine, chlorpromazine, clozapine, and aripiprazole) [6], which resulted in this particular variant being included in some clinically available pharmacogenetic testing panels. When information across all studies is taken into consideration, the composite odds ratio from meta-analysis is 0.65 (95% CI = 0.43 to 0.97), demonstrating the odds of response to antipsychotics are approximately 35% less in patients with the ‘risk’ variant (rs1799732). While research studies suggest that *DRD2* genetic variants may contribute to differences in antipsychotic response, the clinical utility of this information is yet unclear.

Our patient’s different response to two aripiprazole injection formulations with similar daily doses (from injection: approximately 19 mg/d vs. 21 mg/d) is intriguing. One possible explanation may involve differences in pharmacokinetic profiles between two aripiprazole injection formulations. The pharmacokinetic parameters for these two formulations are included in Table 3. At steady state, decline in drug plasma concentration starts approximately 7 days after injection (T_max_ = 6.5 days, see reference [20]) with aripiprazole LAI, whereas the drop is delayed to approximately 21 days after injection (T_max_ = 21.1 days) with aripiprazole lauroxil [8]. This is thought to be from more controlled release of the lauroxil formulation after injection via utilization of LinkeRx technology [21, 22]. Given the lack of head to head data, it is difficult to directly compare pharmacokinetics of the two products, although less fluctuation in aripiprazole and its active metabolite levels from slower dissolution rate of aripiprazole lauroxil may explain why our patient does not exhibit wearing-off symptoms anymore with this product.

**Table 3:** T_max_ and T_1/2_ (mean (SD)) of aripiprazole after administration of long acting injectable aripiprazole (Abilify Maintena®) and aripiprazole lauroxil (Aristada®)

	After single gluteal injection of aripiprazole 400 mg equivalents		After 4th gluteal injection of aripiprazole 300 mg equivalents^a^	
	Aripiprazole LAI	Aripiprazole lauroxil	Aripiprazole LAI	Aripiprazole lauroxil
T_max_ (day)	22 (4–22)^b^	44.0 (11.7)	6.5 (0.5–21.2)^b^	21.1
T_1/2_ (day)	10.5 (3.9)	18.0 (4.8)	29.9 (8.0)	29 (6)

^a^Pharmacokinetic parameters at steady-state. Data for the 400 mg equivalents is not available for aripiprazole lauroxil. For the purpose of comparison between two agents, data for the 300 mg equivalents is included

^b^Reported as median (range)

To date only a few polymorphisms in *DRD2* (i.e. rs1800497 and rs1799732) are currently available as clinical tests [5], and even for the variants that are included on commercially available pharmacogenetic panels, literature focusing on how best to use results from those variants (such as an association of the variants with need for altered dosing strategies) is lacking. One recently published study showed that antipsychotic dose requirement is more likely to be increased over time in schizophrenia patients with the -141C Del allele compared to the Del non-carriers. Although it was a preliminary study with a relatively small sample size, the findings suggested a relationship between the Del allele and need for higher antipsychotic doses in chronic schizophrenia patients [23]. In addition, the need for altered dosing strategies may be consistent with previous finding indicating that patients with schizophrenia who carry the -141C Del allele had significantly longer time to achieve sustained clinical response to antipsychotic medications compared to those with Ins/Ins homozygous [24]. In this study, authors suggested a possibility that lower brain D2 receptor density in patients having Ins/Ins genotype may make antipsychotic agents more efficient at reaching therapeutic levels of blockade compared to Del carriers.

An additional consideration is that the allele frequencies of variants in *DRD2* differ across populations, with the -141C Del variant known to be more common in persons of African Ancestry (up to approximately 60%), such as our patient [25, 26]. While extensive differences in antipsychotic responsiveness are not usually observed across race groups, one previous study showed that the proportion of patients being prescribed antipsychotic dosages above the recommended range (300–1000 chlorpromazine equivalents) was 10% higher in African Americans compared to Caucasians (*p*-value = 0.003). However, the use of depot injections may confound the association between higher antipsychotic dosing and patient race in this study [27]. Two other studies have also reported that African Americans were more likely to be on a high dose (>1000 chlorpromazine equivalents) than Caucasians [28, 29], although Segal et al. concluded the results may be due to less treatment engagement with African American patients [29]. As confounding factors (i.e. use of LAI antipsychotics or treatment engagement) made it difficult to accurately assess this relationship, more research is needed to examine how race or ancestry may influence antipsychotic dosing patterns. One hypothesis for further investigation is that higher allele frequency of the -141C Del variant in persons of African Ancestry may, at least in part, explain higher antipsychotic doses in these patients.

## Conclusions

We report an African American patient who exhibited wearing-off symptoms with aripiprazole LAI and then benefited from changing to the aripiprazole lauroxil formulation. Pharmacogenetic testing ruled out genetic alteration that may influence drug metabolism, but noted that the patient carries a *DRD2* -141C Del polymorphism (rs1799732), which may alter D2 density in the brain. It is important to clarify that co-occurrence of this variant in an individual having a history of antipsychotic nonresponse and increased dosing requirements does not imply causation. However, previous meta-analyses have associated this polymorphism with reduced response, and it is included on some psychiatry focused pharmacogenomic panels for this reason, but without guidance or supporting evidence to suggest what to do for carriers. The importance of presenting this case is therefore two-fold. The first is that some patients with partial response or symptom recurrence towards the end of the usual dosing interval for aripiprazole LAI may benefit from switching to an alternative formulation. The reasons for this are unclear and warrant further investigation. Second, our case suggests that some individuals may be sensitive to the pharmacokinetic differences between the aripiprazole LAI and lauroxil formulations at similar overall exposures, but the biological reasons for this are unknown. One hypothesis for further exploration is that genetic variation resulting in altered D2 expression or function may result in altered sensitivity to antipsychotic medications, requiring different dosing or drug selection strategies.

## Abbreviations

CYP: Cytochrome P450
*DRD2*: Dopamine-D2 receptor gene
EM: Extensive metabolizer
FDA: U.S. Food and Drug Administration
GWAS: Genome-wide association studies
IM: Intramuscularly
LAI: Long-acting injectable
PM: Poor metabolizer
UM: Ultra-rapid metabolizer
